# Application of PBL combined with CBL teaching method in clinical teaching of vascular surgery

**DOI:** 10.1371/journal.pone.0306653

**Published:** 2024-08-15

**Authors:** Mao Zhang, Wei Hu

**Affiliations:** Department of Vascular Surgery, Sichuan Provincial People’s Hospital, University of Electronic Science and Technology of China, Chengdu, China; Medical College of Wisconsin - Central Wisconsin Campus, UNITED STATES

## Abstract

As medical education evolves, Problem-Based Learning (PBL) and Case-Based Learning (CBL) methodologies have emerged as vital pedagogical tools. This study aims to delve into the application and effectiveness of a hybrid PBL-CBL approach in clinical teaching for vascular surgery. By conducting a comparative analysis through concrete teaching practices, this research evaluates the impact on students’ clinical knowledge retention, advancement in clinical reasoning skills, and proficiency in resolving real-world clinical challenges. The findings reveal that the integrated PBL-CBL methodology substantially enhances learning outcomes in vascular surgery clinical teaching, ultimately fostering significant development in students’ comprehensive clinical abilities.

## 1. Introduction

With the continuous evolution of modern medical education, traditional teaching models are gradually giving way to more dynamic, student-centered teaching methods [1]. Among these new methods, Problem-Based Learning (PBL) and Case-Based Learning (CBL) stand out particularly for their significant effects in cultivating students’ active learning abilities, critical thinking, and problem-solving abilities. The PBL teaching method emphasizes student-centeredness and promotes independent learning and team collaboration by solving real or simulated problems [2]. The CBL teaching method helps students establish connections between theory and practice through in-depth analysis of specific cases, enhancing their clinical decision-making abilities [3].

Given the intricate nature of vascular surgery as a highly specialized medical domain, it is imperative for students to possess both robust theoretical foundations, and superior clinical skills along with sound decision-making abilities. Consequently, it becomes crucial to explore and implement more efficient teaching methodologies to augment the effectiveness of vascular surgery education. The PBL combined with CBL teaching method combines the advantages of the two teaching methods, aiming to improve students’ comprehensive abilities through problem-solving and case analysis, enabling them to make quick and accurate judgments and decisions when facing complex vascular surgery problems.

The main purpose of this study is to explore the application effects of the PBL combined with CBL teaching method in clinical teaching of vascular surgery. We hope to assess the effectiveness of PBL combined with CBL in improving students’ mastery of vascular surgery knowledge, enhancing clinical thinking abilities, and solving practical clinical problems by comparing traditional teaching methods.

From a broader perspective, the significance of this study lies not only in improving the teaching quality of vascular surgery but also in providing a new and more effective teaching model for modern medical education. By implementing the PBL combined with CBL teaching method, we expect to cultivate more medical talents with critical thinking, independent learning abilities, and excellent clinical decision-making abilities. Additionally, the results of this study can serve as a reference for teaching reforms in other disciplines, promoting continuous innovation and development of the entire medical education system.

## 2. Experimental subjects and method

### 2.1 Experimental subjects

80 medical students who studied in the Vascular Surgery Department of Sichuan Provincial People’s Hospital from March 1, 2022, to March 1, 2024, were randomly selected as the subjects of the study. Using stratified randomized sampling, the students were divided into a PBL + CBL teaching model group (n = 40) and a traditional teaching model group (n = 40). The PBL + CBL teaching model group had a comprehensive score of (80.95 ± 1.011) upon admission, while the traditional teaching model group had a comprehensive score of (81.93 ± 0.8215). There was no statistically significant difference in baseline data between the two groups of students upon admission (p > 0.05), indicating that the groups were comparable at baseline. To homogenize the data, we have already ensured that the participants in both groups had similar baseline scores and no statistically significant differences in gender, age, or other potentially relevant factors.

### 2.2 Method

#### 2.2.1 Teaching method design

This study meets the ethical requirements of the Research Ethics Committee of Sichuan Provincial People’s Hospital and conforms to the Helsinki Declaration.

In clinical teaching of vascular surgery, we designed a teaching method combining PBL with CBL(Table 1). Firstly, we selected representative and educational vascular surgery diseases that cover common peripheral vascular diseases and their treatment strategies. Then, based on these diseases, we designed guiding questions aimed at stimulating students’ interest and motivation to explore. These questions not only involve basic theoretical knowledge but also include practical applications such as clinical decision-making and treatment plan selection. This is followed by group discussion and deeper learning through real cases. This teaching model is distinctly different from the traditional teaching model that is predominantly teacher-centered, textbook-oriented, and classroom lecture-based.

**Table 1:** Teaching method design of PBL combined with CBL.

Disease Name	PBL Phase:	CBL Phase:
Abdominal Aortic Aneurysm	1. Raise questions: What is an abdominal aortic aneurysm? What are its typical clinical manifestations? 2. Students are divided into groups and search for relevant information through libraries, the Internet, and other resources. 3. Discuss in groups, organize and share information about the pathogenesis, risk factors, diagnosis, and treatment methods of abdominal aortic aneurysms.	1. Provide a real case of abdominal aortic aneurysm. 2. Students analyze the diagnosis basis, treatment plan, and possible complications based on the case. 3. Simulate the development of a surgical plan and discuss postoperative management and follow-up.
Aortic Dissection	1. Raise questions: What is the pathological mechanism of aortic dissection? How is it usually diagnosed? 2. Students self-study and discuss the clinical manifestations, diagnostic methods, and treatment strategies of aortic dissection. 3. The teacher guides the discussion to deepen the understanding of aortic dissection.	1. Provide a typical case of aortic dissection. 2. Students analyze the case and explore emergency treatment measures and long-term treatment plans. 3. Role-play, simulating the diagnosis and treatment process in an emergency situation.
Varicose Veins of the Lower Extremities	1. Explore the question: What are the main causes and clinical manifestations of varicose veins in the lower extremities? 2. Students learn about the pathogenesis and treatment options of varicose veins of the lower extremities through self-study and group discussion. 3. The teacher summarizes, emphasizing the importance of prevention and management.	1. Provide case information on varicose veins of the lower extremities. 2. Students discuss treatment plans based on the case, including conservative treatment and surgical treatment. 3. Simulate the surgical treatment process to allow students to master related techniques.
Arteriosclerosis Obliterans of the Lower Extremities	1. Raise questions: How does arteriosclerosis obliterans of the lower extremities affect blood supply? What are the treatment methods? 2. Students learn about the pathological process and treatment strategies of the disease by consulting relevant materials. 3. Discuss in groups to form a comprehensive understanding of the disease.	1. Show a case of arteriosclerosis obliterans of the lower extremities. 2. Students discuss treatment plans around the case, including drug treatment and interventional therapy. 3. Simulate the interventional surgery process to deepen students’ understanding of the treatment process.
Deep Venous Thrombosis of the Lower Extremities	1. Raise questions: What are the risk factors for deep venous thrombosis of the lower extremities? How to diagnose and treat it? 2. Students self-study and discuss the basic knowledge of deep venous thrombosis of the lower extremities. 3. The teacher comments and emphasizes the importance of prevention and early treatment.	1. Provide case information on deep venous thrombosis of the lower extremities. 2. Students analyze the diagnosis basis, treatment plan, and preventive measures based on the case. 3. Simulate the anticoagulation and thrombolytic therapy process in an emergency situation.

#### 2.2.2 Teaching case analysis

Taking a case of lower extremity arteriosclerotic occlusive disease as an example:

1. Preliminary Preparation
  Case selection: Choose a typical case of a patient with lower extremity arteriosclerotic occlusive disease. The patient should have a clear diagnosis, treatment process, and prognosis.
2. Teaching goal setting:
  - Understand the etiology and pathophysiology of lower extremity arteriosclerotic occlusive disease.
  - Understand the clinical manifestations, diagnosis, and differential diagnosis of the disease.
  - Familiar with treatment principles, methods, and possible complications.
  - Understand the importance of patient education and psychological support.

2.2.2.1. *PBL stage*

- Problem Design:
  Problem 1: What are the causes of lower extremity arteriosclerotic occlusive disease?
  Problem 2: What are the typical clinical manifestations of this disease?
  Problem 3: How is lower extremity arteriosclerotic occlusive disease diagnosed? What diseases need to be differentiated?
  Problem 4: What are the main treatment methods for this disease currently?
- Independent Learning: Students independently search for relevant information and organize it based on the problems.
- Group Discussion: Students discuss in groups, share the information they find, and conduct in-depth analysis and answers to each question.
- Achievement Display: Each group selects a representative to present the discussion results to the whole class.

2.2.2.2. *CBL stage*

- Case Introduction: The teacher introduces the selected case to the students in detail, including the patient’s medical history, physical examination, imaging and laboratory test results, diagnosis, and treatment process.
- Case Analysis: Students analyze the case, discuss the basis for diagnosis, the choice of treatment plan and its reasons, and prognosis evaluation.
- Role Play: Students conduct role-playing of medical communication, treatment decision-making, and other scenes in groups to simulate actual clinical operations.
- Summary Feedback: The teacher comments on the students’ discussion and role-playing, emphasizing key points and issues that should be paid attention to in clinical practice.

Through this PBL combined with CBL teaching method, students can not only deeply understand the knowledge about the disease and treatment strategies of vascular surgery but also improve their clinical skills in practice. This teaching method effectively improves students’ interest and enthusiasm in learning, laying a solid foundation for cultivating high-quality medical talents.

#### 2.2.3 Teaching effectiveness evaluation

2.2.3.1. *Evaluation methods and indicator system*. To comprehensively evaluate the effectiveness of PBL combined with CBL in clinical teaching of vascular surgery, we adopted multiple evaluation methods and constructed a comprehensive indicator system.

The specific methods are as follows:

- Theoretical Test: Design a test paper covering vascular surgery knowledge points to test students’ mastery of basic knowledge.
- Clinical Skill Assessment: Simulate real clinical scenarios to assess students’ clinical operation skills and decision-making abilities.
- Group Discussion Evaluation: Observe and record students’ performance in group discussions, including the number of speeches, content quality, and team collaboration ability.
- Questionnaire Survey: Collect data on students’ satisfaction with teaching methods and self-perceived learning effects through questionnaire surveys.

The indicator system includes the following aspects:

- Knowledge Mastery Degree: Measured by theoretical test scores.
- Clinical Skill Level: Assessed by the score of clinical skill assessment.
- Team Collaboration Ability: Determined based on performance evaluation in group discussions.
- Learning Attitude and Interest: Reflected through relevant topics in the questionnaire.

2.2.3.2. *Comparative analysis of teaching effects*. At the end of the rotation, we conducted a comparative analysis of the teaching effects between students who received PBL combined with CBL teaching and those who received traditional teaching methods. A unified comprehensive assessment and questionnaire survey were conducted. We used a 100-point scale to measure theoretical knowledge, practical skills, and other aspects. Finally, the statistical software GraphPad Prism was used for data analysis and processing. Continous variable are represented by means and SD, categorical variables as percentages. Continous variables compared by T-test or difference in means and categorical by chi squared. A p-value < 0.05 was considered statistically significant.

## 3. Result

### 3.1 The findings indicated that

- In terms of knowledge mastery, students in the PBL combined with CBL teaching group had significantly higher theoretical test scores than those in the traditional teaching group(See Table 2).
- In terms of clinical skill level, students in the PBL combined with CBL teaching group demonstrated stronger practical operation abilities and clinical decision-making abilities in the clinical skill assessment(See Table 2).
- Regarding team collaboration, students in the PBL combined with CBL teaching group exhibited a higher level of team collaboration and a more active participation attitude in group discussions(See Table 3).

**Table 2:** Comparing students’ theoretical and practical performance under two teaching modes.

Group category	Theoretical knowledge	Practical skills
CBL+PBL teaching group(n = 40)	85.50 ± 1.485	90.05 ± 0.7052
Traditional teaching group(n = 40)	75.75 ± 1.195	84.20 ± 0.8299
t	t = 5.115	t = 5.372
p	< 0.0001	< 0.0001

**Table 3:** Comparison of students’ team collaboration ability between two teaching groups.

Group Category	Total number	Excellent	Good	Pass	Fail
CBL+PBL teaching group	40	18	12	9	1
Traditional teaching group	40	9	14	14	3
χ2					4.9241
P					0.0265

### 3.2 Student feedback and suggestion collection

To gain a deeper understanding of students’ views and suggestions on the combined PBL and CBL teaching method, we conducted a questionnaire survey (See Table 4). Most students indicated that this teaching approach had enhanced their interest and enthusiasm for learning, enabling them to comprehend and grasp the knowledge of vascular surgery more deeply. Meanwhile, students also provided valuable feedback, such as their desire for teachers to present more authentic clinical cases and offer more guidance and feedback during discussions. We will carefully consider and incorporate these suggestions to further refine our teaching methods and improve the quality of education.

**Table 4:** Two groups of students’ satisfaction survey forms.

Group Category	CBL+PBL teaching group (n = 40)	Traditional teaching group (n = 40)	Difference between means	R square	P
Teaching satisfaction	90.23 ± 0.7865	75.73 ± 1.170	14.50 ± 1.410	0.5756	< 0.0001
Autonomous learning ability	90.55 ± 0.8571	77.58 ± 1.117	12.98 ± 1.408	0.5212	< 0.0001
Ability to identify and analyze problems	88.83 ± 0.8548	78.15 ± 1.223	10.68 ± 1.492	0.3961	< 0.0001
Ability to independently solve problems	88.73 ± 0.9412	76.05 ± 1.265	12.68 ± 1.577	0.4531	< 0.0001
Case analysis ability	89.08 ± 0.9570	75.95 ± 1.160	13.13 ± 1.504	0.4940	< 0.0001
Clinical practice ability	88.53 ± 0.8608	75.90 ± 1.067	12.63 ± 1.371	0.5209	< 0.0001
Team collaboration ability	90.55 ± 0.9240	78.25 ± 1.318	12.30 ± 1.610	0.4281	< 0.0001

## 4. Discussion

### 4.1 Theoretical basis of PBL combined with CBL teaching

PBL combined with CBL teaching integrates the advantages of both teaching methods, aiming to comprehensively improve students’ abilities through problem-solving and case analysis [4]. In this combined teaching model, students need to actively explore and learn through real problems, and they also need to deeply analyze cases to exercise clinical decision-making and problem-solving abilities.

The theoretical basis of PBL combined with CBL teaching mainly includes the following points [5]:

1. Constructivist Learning Theory: This theory maintains that learning is a process of active knowledge construction by learners, rather than passive acceptance [6]. PBL combined with CBL teaching emphasizes student initiative and independent exploration, which aligns with the constructivist view of learning.
2. Cooperative Learning Theory: This theory emphasizes cooperation and mutual assistance among learners, believing that cooperative learning can promote deep understanding of knowledge and mastery of skills [7]. The group cooperation and discussion in PBL combined with CBL teaching reflect the concept of cooperative learning.
3. Situated Cognition Theory: This theory believes that knowledge is constructed and applied in specific contexts [8]. PBL combined with CBL teaching creates learning contexts through real problems and cases, helping students apply knowledge in practical situations.

In summary, the theoretical basis of PBL combined with CBL teaching integrates various advanced educational concepts and methods, aiming to comprehensively improve students’ abilities through problem-solving and case analysis.

### 4.2 Advantages of combined PBL and CBL teaching

The combination of PBL and CBL teaching demonstrates significant advantages in clinical teaching of vascular surgery. Firstly, this student-centered approach greatly enhances students’ initiative and participation through problem-solving and case analysis. When faced with real or simulated clinical problems, students are required to actively search for information, analyze the situation, and propose solutions. This process cultivates students’ independent learning skills, while simultaneously refining their clinical thinking and problem-solving abilities.

Secondly, the integration of PBL and CBL facilitates the connection between theory and practice. By analyzing authentic clinical cases, students can apply theoretical knowledge to practical situations, deepening their understanding and retention of the material. This teaching method also acquaints students with the complexity and variability of actual clinical work, laying a solid foundation for their future clinical practice.

Lastly, the combined PBL and CBL approach fosters students’ teamwork and collaboration skills. During group discussions and case analyses, students need to cooperate and jointly solve problems, which not only enhances their communication abilities but also cultivates a spirit of teamwork, crucial for medical students’ future professional development.

### 4.3 Existing issues and improvement measures

Despite the numerous advantages of combined PBL and CBL teaching, there are still some challenges in its implementation. Firstly, this teaching method demands a high level of expertise and teaching skills from instructors to effectively guide students in discussions and analyses. Therefore, it is necessary to strengthen teacher training and selection to improve their teaching proficiency and professional quality. Secondly, the integration of PBL and CBL demands students to dedicate considerable time and energy towards self-directed study and collaborative discussions, which can be challenging for those with a weaker academic foundation or limited self-discipline. To address this, teachers can provide additional learning resources and guidance, assisting students in establishing effective learning methods and habits.

### 4.4 Implications for future medical education

The successful application of PBL combined with CBL teaching in clinical vascular surgery education provides valuable insights for future medical education. Firstly, medical education should place greater emphasis on student initiative and participation, stimulating their interest and motivation through challenging and engaging teaching tasks. Secondly, it is essential to strengthen the integration of theory and practice in medical education. By introducing more authentic clinical cases and practical opportunities, students can transform theoretical knowledge into practical skills, enhancing their clinical practice abilities. Finally, medical education should cultivate students’ teamwork and communication skills. In future medical work, doctors need to communicate and collaborate effectively with patients, colleagues, and other medical team members. Therefore, medical education should focus on developing students’ communication skills and teamwork spirit, improving their overall quality and professional development capabilities.

### 4.5 The limitations of our study

Our study still has some limitations, which we will discuss from three aspects.

Teacher factor: Although some measures have been taken to reduce potential biases from teachers towards the two groups of students, since both groups share the same teachers, teachers may unintentionally provide more attention or guidance to one group, thereby affecting the learning outcomes of both groups.

Student group characteristics: While the study has attempted to ensure that there are no significant differences in baseline data such as gender and age between the two groups of students, there may still be differences in other characteristics of the student groups, such as academic background, learning motivation, and individual abilities. These differences may have an impact on the study results.

Consistency in implementing teaching methods: The implementation of PBL and CBL teaching methods requires certain skills and experience. Although teachers may have received relevant training, in actual teaching, different teachers may have different understandings and implementations of the teaching methods, which may affect the evaluation of teaching effects.

## 5. Conclusion

### 5.1 Summary of the study

This study explored the application effects of PBL combined with CBL teaching in clinical vascular surgery education. Through the implementation of this teaching method, we observed significant improvements in students’ knowledge mastery, clinical skills, and problem-solving abilities. The student-centered approach of PBL combined with CBL effectively promotes active learning, critical thinking, and teamwork abilities through the analysis and discussion of real problems and cases. Simultaneously, this teaching method enhances the connection between theory and practice, making students more adept at solving practical clinical problems. The research results indicate that PBL combined with CBL is a highly effective teaching method, particularly suited for specialized medical fields like vascular surgery. It not only enhances teaching quality but also lays a solid foundation for students’ future professional development.

### 5.2 Prospects for future research

Although this study has initially confirmed the effectiveness of PBL combined with CBL in clinical vascular surgery education, there are still many aspects worthy of further exploration. Firstly, in-depth research can be conducted on the application effects of PBL combined with CBL in different medical fields to verify applicability across the board. Additionally, comparative studies can be performed on the long-term outcomes of PBL combined with CBL and other teaching methods (such as traditional teaching, PBL alone, or CBL alone) to provide a more comprehensive evaluation of teaching effectiveness. Secondly, future research can focus on the impact of PBL combined with CBL on students’ long-term career development. For instance, observing the performance of students who have received this teaching method in clinical practice, as well as their job satisfaction and sense of accomplishment. Finally, with the continuous development of educational technology, combining PBL with CBL and advanced technologies like online education and virtual reality to improve teaching effectiveness and efficiency is also a worthwhile research direction.

In summary, PBL combined with CBL holds vast application prospects and research value in clinical vascular surgery education. Future research should continue to deepen and refine this teaching method to better cultivate high-quality and capable medical talents.

## References

[1] Kim KJ, Hwang JY. Characteristics of medical teachers using student-centered teaching methods. Korean J Med Educ. 2017; 29: 187–191. doi: 10.3946/kjme.2017.64 28870021 PMC5583433

[2] Lim WK. Problem Based Learning in Medical Education: Handling Objections and Sustainable Implementation. Adv Med Educ Pract. 2023; 14: 1453–1460. doi: 10.2147/AMEP.S444566 38164409 PMC10758192

[3] Cen XY, Hua Y, Niu S, Yu T. Application of case-based learning in medical student education: a meta-analysis. Eur Rev Med Pharmaco. 2021; 25: 3173–3181. doi: 10.26355/eurrev_202104_25726 33928603

[4] Zhao W, He L, Deng W, Zhu J, Su A, Zhang Y. The effectiveness of the combined problem-based learning (PBL) and case-based learning (CBL) teaching method in the clinical practical teaching of thyroid disease. Bmc Med Educ. 2020; 20: 381. doi: 10.1186/s12909-020-02306-y 33092583 PMC7583209

[5] Yang W, Li H, Su A, Ding L. Application of problem based learning (PBL) and case based learning (CBL) in the teaching of international classification of diseases encoding. Sci Rep-Uk. 2023; 13: 15220. doi: 10.1038/s41598-023-42175-1 37709817 PMC10502146

[6] Hrynchak P, Batty H. The educational theory basis of team-based learning. Med. Teach. 2012; 34: 796–801. doi: 10.3109/0142159X.2012.687120 22646301

[7] Scager K, Boonstra J, Peeters T, Vulperhorst J, Wiegant F. Collaborative Learning in Higher Education: Evoking Positive Interdependence. Cbe-Life Sci Educ. 2016; 15. doi: 10.1187/cbe.16-07-0219 27909019 PMC5132366

[8] Wang M, Chen X, Yang Y, Wang H, Yan Y, Huang X, et al. Effect evaluation of case-based learning with situated cognition theory on competence training for student nurses in pediatric surgery. Heliyon. 2023; 9: e13427. doi: 10.1016/j.heliyon.2023.e13427 36820019 PMC9937989

